# Robust network structure of the Sln1-Ypd1-Ssk1 three-component phospho-relay prevents unintended activation of the HOG MAPK pathway in *Saccharomyces cerevisiae*

**DOI:** 10.1186/s12918-015-0158-y

**Published:** 2015-03-25

**Authors:** Joseph P Dexter, Ping Xu, Jeremy Gunawardena, Megan N McClean

**Affiliations:** Lewis-Sigler Institute for Integrative Genomics, Princeton University, Princeton, NJ USA; Department of Chemistry, Princeton University, Princeton, NJ USA; Department of Systems Biology, Harvard Medical School, Boston, MA USA; Department of Biomedical Engineering, University of Wisconsin-Madison, Madison, WI USA

**Keywords:** HOG pathway, Osmotic stress, Histidine kinase, Robustness, Mathematical modeling, Invariants

## Abstract

**Background:**

The yeast *Saccharomyces cerevisiae* relies on the high-osmolarity glycerol (HOG) signaling pathway to respond to increases in external osmolarity. The HOG pathway is rapidly activated under conditions of elevated osmolarity and regulates transcriptional and metabolic changes within the cell. Under normal growth conditions, however, a three-component phospho-relay consisting of the histidine kinase Sln1, the transfer protein Ypd1, and the response regulator Ssk1 represses HOG pathway activity by phosphorylation of Ssk1. This inhibition of the HOG pathway is essential for cellular fitness in normal osmolarity. Nevertheless, the extent to and mechanisms by which inhibition is robust to fluctuations in the concentrations of the phospho-relay components has received little attention.

**Results:**

We established that the Sln1-Ypd1-Ssk1 phospho-relay is robust—it is able to maintain inhibition of the HOG pathway even after significant changes in the levels of its three components. We then developed a biochemically realistic mathematical model of the phospho-relay, which suggested that robustness is due to buffering by a large excess pool of Ypd1. We confirmed experimentally that depletion of the Ypd1 pool results in inappropriate activation of the HOG pathway.

**Conclusions:**

We identified buffering by an intermediate component in excess as a novel mechanism through which a phospho-relay can achieve robustness. This buffering requires multiple components and is therefore unavailable to two-component systems, suggesting one important advantage of multi-component relays.

**Electronic supplementary material:**

The online version of this article (doi:10.1186/s12918-015-0158-y) contains supplementary material, which is available to authorized users.

## Background

**Figure 1:**
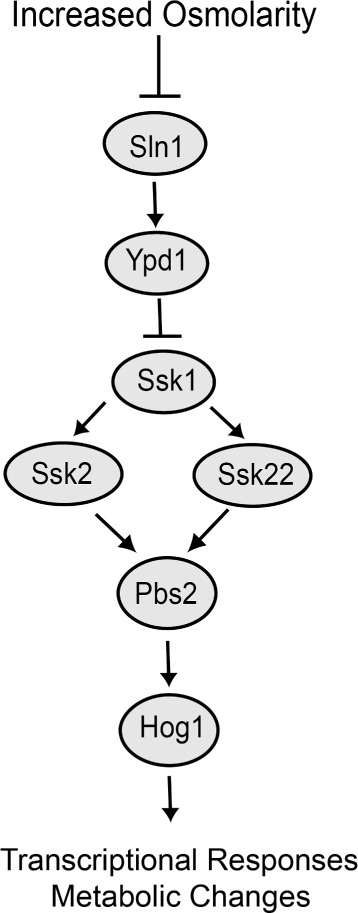
**Structure of the Sln1-Ypd1-Ssk1 relay and the HOG MAPK pathway.** Under normal growth conditions, Sln1 activates Ypd1, which in turn inhibits Ssk1. Increased osmolarity inhibits Sln1, resulting in activation of Ssk1 and of the MAP kinase Hog1 through the upstream MAP3Ks Ssk2 and Ssk22 and the MAP2K Pbs2. Activated Hog1 regulates transcriptional and metabolic changes within the cell that increase production and retention of glycerol.

The high-osmolarity glycerol (HOG) pathway (Figure 1) of the budding yeast *Saccharomyces cerevisiae* mediates cellular response to increased external osmolarity [1,2]. A key component of the HOG pathway is a mitogen-activated protein (MAP) kinase cascade. Within the kinase cascade, the MAP3Ks Ssk2 and Ssk22 phosphorylate the MAP2K Pbs2, which in turn phosphorylates the MAP kinase Hog1. Phospho-Hog1 then regulates transcriptional and metabolic changes that increase production and accumulation of the compatible solute glycerol. Mounting a rapid response to increased osmolarity is essential to yeast survival [3,4]. Accordingly, *S. cerevisiae* can activate the HOG pathway within one minute of experiencing an osmotic shock [5]. Yeast can also effectively respond to rapid periodic oscillations (with frequencies up to 0.0046 Hz) between low and high external osmolyte concentrations [3].

**Figure 2:**
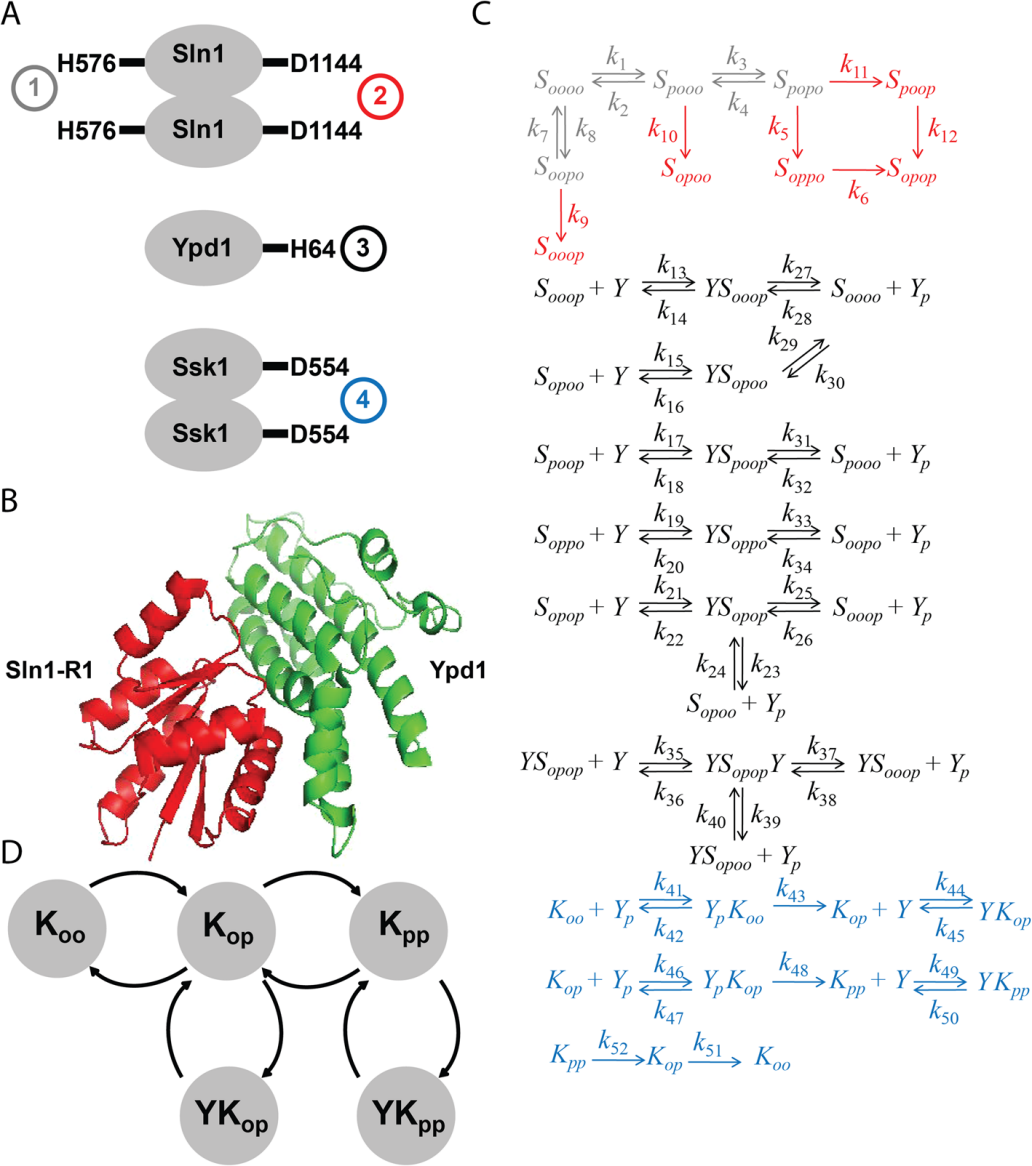
**Biochemically realistic model of the HOG pathway three-component phospho-relay in** ***S. cerevisiae*** **.** **A** Cartoon diagram of the phospho-relay indicating the quaternary structure of each component and the four relevant phosphorylation sites. Phospho-transfer proceeds from Sln1 (after autophosphorylation on H576 and transfer to D1144) to Ypd1 to Ssk1, as indicated by the numbers in circles. **B** Crystal structure of Ypd1 (green) in complex with the response regulator domain of Sln1 (Sln1-R1, red). Drawn from data presented in [18]. **C** Reaction network diagram describing our model of the phospho-relay. The network includes nearly all possible interactions between the three proteins subject to the biochemical assumptions outlined in the main text. For clarity, the reaction network is color-coded to indicate the groups of reactions involved in each phosphorylation event. *S* denotes Sln1, *Y* denotes Ypd1, and *K* denotes Ssk1. Phosphorylated residues are denoted by *p*, unphosphorylated residues by *o*. **D** Directed graph describing the subnetwork involving phosphorylation and dephosphorylation of Ssk1. The graph contains four loops that are connected as a branched tree.

Despite its importance during periods of increased osmolarity, unintended activation of the HOG pathway during growth in normal osmolarity conditions is severely deleterious [6,7]. The Sln1-Ypd1-Ssk1 three-component phospho-relay is responsible for maintaining inactivation of the HOG pathway under normal conditions. This three-component phospho-relay is a variant of the two-component signaling systems used by many prokaryotes for osmoregulation, chemotaxis, and other key cellular processes. Sln1 is active *in vivo* as a membrane-bound dimer [6]. Under normal osmolarity conditions, Sln1 autophosphorylates on a histidine residue and then irreversibly transfers the phosphate to an aspartate in its response regulator (RR) domain (Figure 2A). Aspartate-phosphorylated Sln1 binds to the histidine-containing phospho-transfer (HPt) protein Ypd1 and reversibly transfers its phosphate to Ypd1 (Figure 2B). Finally, phospho-Ypd1 transfers its phosphate to dimeric Ssk1, preventing it from interacting with Ssk2 (or the functionally redundant Ssk22) and inhibiting HOG pathway activity [5,8,9]. The sequence of phosphate transfers in the three-component relay is summarized in Figure 2A. In response to osmotic shock, the phospho-relay is inactivated, and Ssk1 is rapidly dephosphorylated through an as-yet unknown mechanism. Unphosphorylated Ssk1 then activates Ssk2 and Ssk22, leading to induction of the HOG pathway [1,2]. It is thus the essential controller of HOG pathway activity, and variations in its concentration could compromise fitness.

There is limited existing experimental evidence that the phospho-relay is able to maintain robust phosphorylation of Ssk1 and inactivation of the HOG pathway despite changes in the levels of some pathway components [7,9,10]. We undertook a comprehensive characterization of the sensitivity of HOG pathway activation to changes in the expression levels of the phospho-relay proteins Sln1, Ypd1, and Ssk1. We systematically under- and overexpressed the three proteins using the GEV artificial induction system, which allows for rapid and nearly gratuitous induced expression of individual yeast genes [11]. We found that the phospho-relay maintains inactivation of the HOG pathway even after moderate perturbation of Sln1, Ypd1, and Ssk1.

We developed a detailed, biochemically realistic mathematical model of the HOG pathway three-component phospho-relay to elucidate the mechanism underlying this robustness (Figure 2C). Our model incorporates extensive structural and mechanistic information about the phospho-relay and considers nearly all possible interactions between the three relay proteins. We used mass-action kinetics and algebraic calculations to characterize the steady-state behavior of the model. Steady-state algebraic models are a useful alternative to existing computational models of the HOG pathway for understanding robust behavior [3,7,12]. Unlike numerical simulations [3,7,12], algebraic manipulations can be done without ever assigning specific values to the parameters (i.e., the rate constants in the reaction network), many of which are difficult or impossible to measure experimentally [13]. This advantage enabled us to design and analyze a more biochemically realistic model. A steady-state approximation is appropriate because previous studies have shown that activation of the HOG pathway does not vary under normal growth conditions [3,4,12,14,15].

Our steady-state analysis predicted that relative levels of dephosphorylated Ssk1 depend solely on Ypd1 levels and that robustness is achieved by maintaining Ypd1 in large excess. We experimentally tested this prediction by perturbing protein expression levels so as to deplete this buffering pool of Ypd1. All such perturbations compromised the ability of the phospho-relay to inhibit the HOG pathway, leading to hyperactivation in normal osmolarity conditions. The presence of a large buffering pool of an intermediate phospho-relay component is a previously underappreciated mechanism for robustness and suggests a possible advantage of a three-component relay over a two-component system.

## Results and discussion

### Inhibition of HOG pathway signaling is robust to moderate overexpression of phospho-relay components

Inappropriate HOG pathway activation during normal osmolarity growth unnecessarily alters transcription and metabolism [7,9,10]. To assess the robustness of HOG pathway inhibition by the three-component phospho-relay, we created strains capable of overexpressing Sln1, Ypd1, and Ssk1 in response to *β*-estradiol. For these overexpression experiments, we used diploid strains homozygous for the GEV artificial transcription factor [11]. GEV consists of the Gal4 DNA-binding domain, the estrogen receptor, and the VP16 activation domain. Upon treatment with the hormone *β*-estradiol, GEV rapidly translocates to the nucleus, where it activates transcription from promoters containing the Gal4 DNA-binding target sequence. The GEV system enables rapid induction of individual yeast genes with limited off-target effects [11]. To make a given phospho-relay gene GEV-inducible, we placed it under the control of the *GAL1* promoter (SLN1/P _*GAL*1_-SLN1, YPD1/P _*GAL*1_-YPD1, and SSK1/P _*GAL*1_-SSK1), as described previously [11].

**Figure 3:**
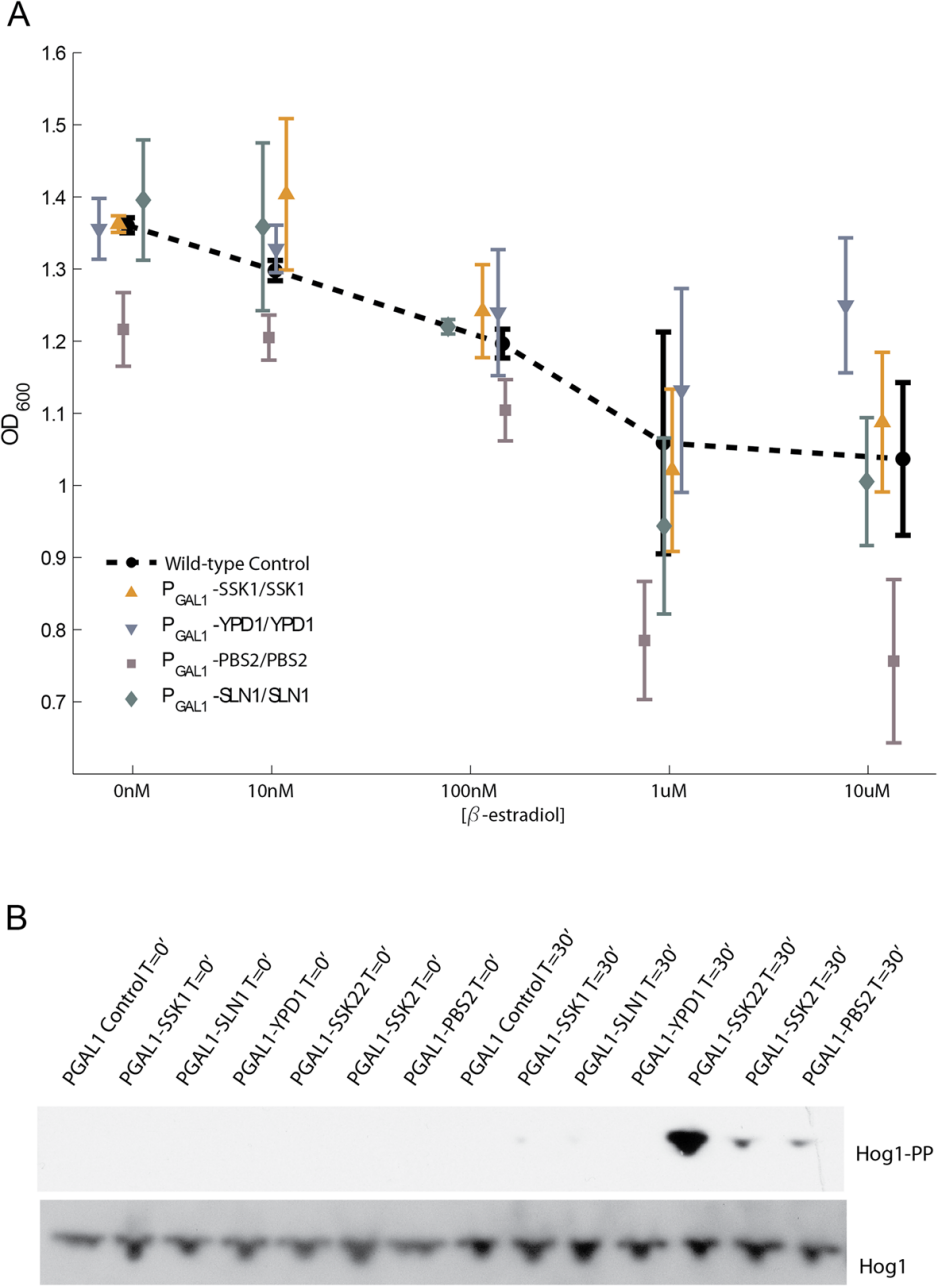
**Moderate overexpression of phospho-relay components does not activate the HOG pathway.** **A** Homozygous GEV diploid strains with a single inducible copy of a phospho-relay gene were grown to saturation in different concentrations of *β*-estradiol. The optical density (OD _600_) after 13 hours of growth is plotted. Overexpression of Pbs2 caused a growth defect at higher concentrations of *β*-estradiol, while no significant growth defects were observed following overexpression of relay components (beyond the defect in the wild-type due to overexpression of Gal4 target genes). Each point represents the mean and standard deviation over four replicates. **B** We assayed for Hog1 phosphorylation using an antibody specific to doubly phosphorylated Hog1 to confirm that moderate overexpression of phospho-relay components does not lead to activation of the HOG pathway. Overexpression of phospho-relay components using a saturating dose (10 *μ*M) of *β*-estradiol did not lead to Hog1 phosphorylation. Overexpression of the positive controls Ssk22, Ssk2, and Pbs2, however, led to clear upregulation of Hog1 phosphorylation after 30 minutes of induction. Total Hog1 is shown as a loading control.

Inappropriate activation of the HOG pathway is known to cause a growth defect [7,9,10]. We therefore measured growth of these GEV strains after induction with *β*-estradiol at a range of concentrations to screen for HOG pathway hyperactivity. A strain carrying an inducible allele of Pbs2 (P _*GAL*1_-PBS2/PBS2) was used as a control because Pbs2 overexpression is known to cause severe growth defects from inappropriate activation of the HOG pathway [7]. As shown in Figure 3A, strains overexpressing Pbs2 exhibited a measurable growth defect, while strains overexpressing components of the phospho-relay (Sln1, Ypd1, and Ssk1) showed no significant change.

We also assayed for Hog1 phosphorylation following GEV induction of phospho-relay components to obtain direct evidence that moderate overexpression does not cause HOG pathway hyperactivation. We used overexpression of Pbs2 and Ssk2 (also known to cause hyperactivation of the HOG pathway [7]) as positive controls. After 30 minutes of GEV induction, there was no detectable increase in Hog1 phosphorylation in strains overexpressing phospho-relay components (Figure 3B). In contrast, Pbs2 and Ssk2 overexpression caused phosphorylation of Hog1. Interestingly, overexpression of the Ssk2 homolog Ssk22 had the strongest effect on Hog1 phosphorylation.

**Figure 4:**
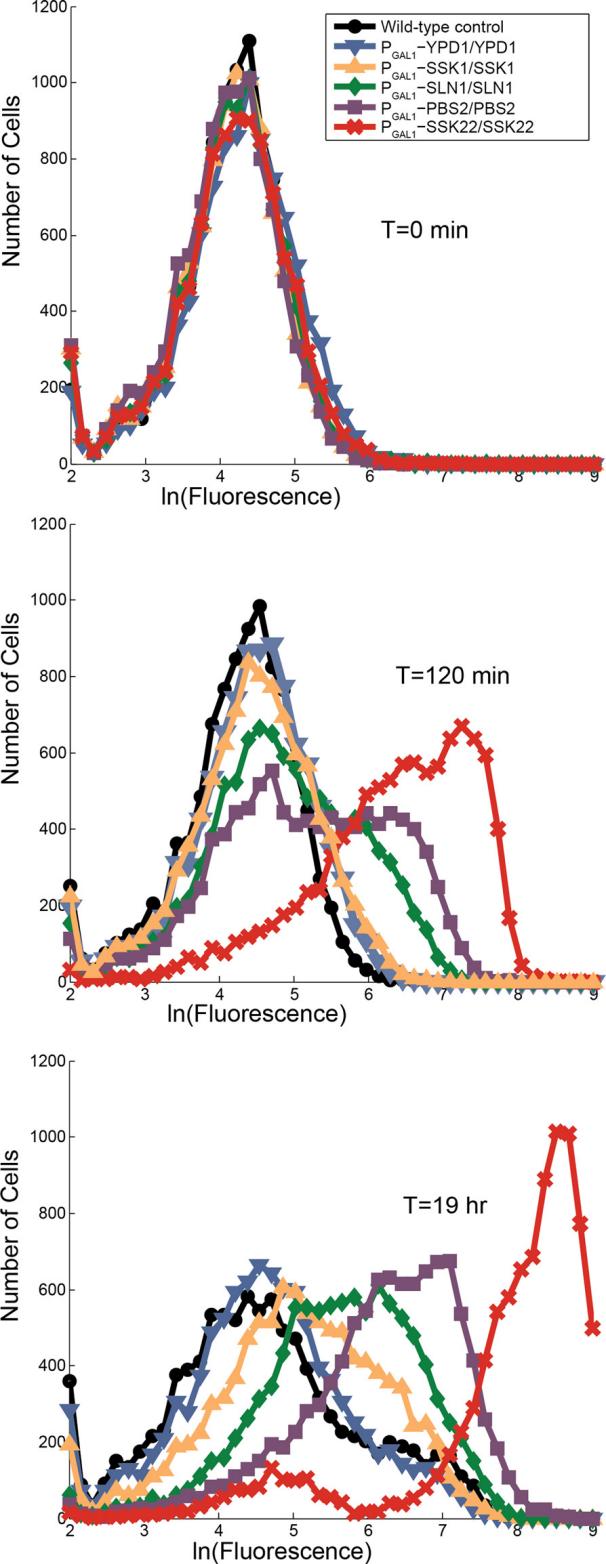
**Moderate overexpression of phospho-relay components causes mild activation of HOG pathway transcriptional targets on long timescales.** *STL1* is a gene induced in response to active Hog1. We replaced one copy of the *STL1* gene with yEVenus in homozygous GEV strains to create a P _*STL*1_-yEVenus transcriptional reporter of HOG pathway activity. We used flow cytometry to analyze yEVenus expression in cells at the start of the experiment and at 2 hours and 19 hours after induction with 10 *μ*M *β*-estradiol. Pbs2 and Ssk22 overexpression caused the strongest activation of the HOG pathway transcriptional reporter. Cell counts are plotted against the natural log of their fluorescence signal [a.u.].

We then constructed diploid strains with a single inducible copy of the HOG phospho-relay gene of interest and a single P _*STL*1_-YFP reporter to assay for HOG pathway transcriptional activity in response to overexpression of relay components. Stl1 is a glycerol/H ^+^ symporter whose expression is strongly upregulated in response to osmotic shock [16]. We overexpressed all three relay components, Pbs2, and Ssk22. Here we used Ssk22 as a control instead of Ssk2 because it showed a strong effect on Hog1 phosphorylation in the previous experiment. After 120 minutes of induction with 10 *μ*M *β*-estradiol, Ssk22 and Pbs2 overexpression led to HOG-dependent transcription from the *STL1* promoter, as indicated by an increase in YFP fluorescence (Figure 4). Over the same period of time, overexpression of the phospho-relay components (Sln1, Ypd1, and Ssk1) caused almost no transcription from the *STL1* promoter. After 19 hours, overexpression of Ssk1 and Sln1 did increase expression of *YFP* from the *P*_*STL*1_-YFP reporter. These effects on longer time scales may have been due to factors beyond Ssk1 and Sln1 overexpression, however, as there was a population expressing YFP even in the control strain at 19 hours.

### Construction of a biochemically realistic model of the HOG pathway phospho-relay

Growth, phosphorylation, and transcriptional measurements of HOG pathway activity all indicated that HOG pathway activation is robust to fluctuations in the Sln1-Ypd1-Ssk1 phospho-relay components. These results prompted us to investigate the mechanistic basis of this robustness using a biochemical model. The reaction network underlying our model (Figure 2C) has 37 nodes and involves 13 species. In this section we discuss the biochemical justification for key assumptions in the model.

There exist high-resolution crystal structures of Ypd1 alone and in complex with the Sln1 receiver domain (Figure 2B) [17,18]. Genetic and biochemical evidence suggest that Sln1 forms an obligate homodimer and that Ypd1 can interact with either half of the dimer [6,19], which implies that formation of a Ypd1-Sln1(dimer)-Ypd1 ternary complex is possible. Accordingly, we include Sln1 in the reaction network as a dimer with four relevant phosphorylation sites. The Sln1 dimer is referred to as *S*_*H*1*D*1*H*2*D*2_, where H1 and D1 denote the phosphorylatable histidine and aspartate residues in one half of the dimer and H2 and D2 denote the corresponding residues in the other half. The dimer is allowed to autophosphorylate on either histidine residue. Phospho-transfer from the histidine to the aspartate in the RR domain is treated as irreversible. A reverse reaction is included with all histidine autophosphorylation steps to account for possible hydrolysis of the phosphate prior to transfer [2,20]. Each half of the dimer is assumed to be independent from the other. Coincident phosphorylation events (e.g., *S*_*OOOO*_ forming *S*_*POPO*_ in one step) are therefore considered to be unlikely and are excluded from the model. Following these assumptions, the model includes nine different forms of free Sln1 that are interconverted as shown in the reaction network.

In the second leg of the phospho-relay, any Sln1 phospho-form with at least one phosphorylated aspartate is allowed to reversibly associate with unphosphorylated monomeric Ypd1 (*Y*) to form a series of binary complexes (*YS*_*OOOP*_, *YS*_*OPOO*_, *YS*_*POOP*_, *YS*_*OPPO*_, and *YS*_*OPOP*_). Sln1-Ypd1 phospho-transfer has been shown to be reversible [21]. As such, all reactions that produce phospho-Ypd1 (*Y*_*P*_) are treated as reversible. No assumption is made about which half Ypd1 binds to in the *YS*_*OPOP*_ binary complex because the halves of the Sln1 dimer are considered to be indistinguishable. Accordingly, the *YS*_*OPOP*_ complex is allowed to form from either *Y*_*P*_+*S*_*OOOP*_ or *Y*_*P*_+*S*_*OPOO*_. Additionally, *YS*_*OPOP*_ can bind to a second *Y* molecule to form a ternary complex (*YS*_*OPOP*_*Y*) that produces *Y*_*P*_ using either of the Sln1 phospho-aspartate residues.

Phospho-Ypd1 then binds to and phosphorylates Ssk1, which is modeled as a dimer with two phosphorylation sites (*K*_*OO*_) [9]. The two monophosphorylated forms of Ssk1, which are known to be fully inactive [9], are assumed to be identical (i.e., *K*_*PO*_=*K*_*OP*_). *Y*_*P*_ can form a complex with *K*_*OO*_, leading to the production of *K*_*OP*_, and in turn *Y*_*P*_ can bind to *K*_*OP*_ and transfer a phosphate to produce *K*_*PP*_. The network includes one further interaction between Ypd1 and Ssk1 deduced from kinetic data. The half-life of phospho-Ssk1 *in vitro* has been measured to be dramatically different with and without the presence of Ypd1 (over 40 hours vs. 13 minutes, respectively), suggesting that Ypd1 binds to *K*_*OP*_ and *K*_*PP*_ to prevent hydrolysis of the phosphate [20,22]. Accordingly, the reaction network includes the reversible formation of dead-end complexes between Ypd1 and phospho-Ssk1. Finally, unstabilized *K*_*PP*_ and *K*_*OP*_ are allowed to lose phosphates via spontaneous hydrolysis. Inclusion of these hydrolysis reactions ensures that there is a complete cycle for Ssk1 modification/demodification and that the system can reach a stable steady state. We emphasize, however, that spontaneous hydrolysis of complexed Ssk1 is likely not the mechanism for rapid dephosphorylation of large quantities of Ssk1 in response to osmotic shock. The mechanism for this rapid activation remains unknown but is irrelevant for our model, which is restricted to yeast growing in steady-state normal osmolarity conditions.

### Derivation of the key invariant

Robust inactivation of the HOG pathway requires that only a small fraction of total Ssk1 (*K*_*T*_) be in the active (*K*_*OO*_) modification form at steady state. The goal of this section is to derive a simple steady-state expression (an invariant) for the ratio of active to total Ssk1. We find an invariant of the form

(1) $$ {}\frac{[\!K_{OO}]}{K_{T}} = \frac{1}{1\!+ \frac{1}{\gamma} \left[\!\alpha [\!Y_{P}] + \beta [\!Y_{P}]^{2} + [\!Y]\left(\alpha^{\prime} [\!Y_{P}] + \beta^{\prime} [\!Y_{P}]^{2}\right)\right]},   $$

where the coefficients are combinations of the rate constants. In this section we derive Eq. 1, and in the following section we discuss experimental tests of its predictions.

The subnetwork involving Ssk1 contains four loops, which are linked in a branched tree (Figure 2D). It is a general feature of such networks that, at steady state, each individual loop is at steady state, irrespective of any other loops in which the components participate [23,24].

If each individual loop is at steady state, then the forward flux through each loop must be balanced by the backward flux, which yields the following four equations:

(2) $$ \begin{aligned} k_{51}\left[K_{OP}\right] & = k_{43}\left[Y_{P}K_{OO}\right] \\ k_{52}\left[K_{PP}\right] & = k_{48}\left[Y_{P}K_{OP}\right] \\ k_{45}\left[{YK}_{OP}\right] & = k_{44}\left[Y\right]\left[K_{OP}\right] \\ k_{50}\left[{YK}_{PP}\right] & = k_{49}\left[Y\right]\left[K_{PP}\right]. \end{aligned}   $$

Because the intermediate complexes *Y*_*P*_*K*_*OO*_ and *Y*_*P*_*K*_*OP*_ are also at steady state, we can write

$$\begin{aligned} k_{43}\left[Y_{P}K_{OO}\right] & = k_{41}\left[Y_{P}\right]\left[K_{OO}\right] - k_{42}\left[Y_{P}K_{OO}\right]\\ k_{48}\left[Y_{P}K_{OP}\right] & = k_{46}\left[Y_{P}\right]\left[K_{OP}\right] - k_{47}\left[Y_{P}K_{OP}\right]. \end{aligned} $$

from which we deduce that

(3) $$ \begin{aligned} \left[Y_{P}K_{OO}\right] &= \frac{k_{41}}{k_{42}+k_{43}}\left[Y_{P}\right]\left[K_{OO}\right]\\ \left[Y_{P}K_{OP}\right] &= \frac{k_{46}}{k_{47}+k_{48}}\left[Y_{P}\right]\left[K_{OP}\right]. \end{aligned}  $$

Substituting Eq. 3 into Eq. 2, we obtain expressions for *K*_*OP*_, *K*_*PP*_, *YK*_*OP*_, and *YK*_*PP*_ in terms of *K*_*OO*_, *Y*, and *Y*_*P*_. From Eq. 3 we already have expressions for *Y*_*P*_*K*_*OO*_ and *Y*_*P*_*K*_*OP*_ in terms of *K*_*OO*_ and *Y*_*P*_. As such, we are able to calculate the total amount of Ssk1 (*K*_*T*_) in terms of just *K*_*OO*_, *Y*, and *Y*_*P*_. We have

$$\begin{array}{@{}rcl@{}} K_{T} &=& \left[K_{OO}\right] + \left[K_{OP}\right] + \left[K_{PP}\right] + \left[Y_{P}K_{OO}\right]\\ &&+ \left[Y_{P}K_{OP}\right] + \left[{YK}_{OP}\right] + \left[{YK}_{PP}\right]. \end{array} $$

Substituting for the individual terms, we obtain

$${} {\fontsize{9.1pt}{9.6pt}\selectfont{\begin{aligned} K_{T} = \left[K_{OO}\right]\left[\!1 + \frac{1}{\gamma} \!\left(\alpha [\!Y_{P}]\! + \beta [\!Y_{P}]^{2} + \left(\alpha^{\prime} [\!Y_{P}] + \beta^{\prime} [\!Y_{P}]^{2}\! \right)\![\!Y]\right)\!\right]\!, \end{aligned}}}  $$

where

$$ \begin{aligned} \alpha &= k_{41}k_{45}k_{50}k_{52}(k_{43}+k_{51})(k_{47}+k_{48}) \\ \beta &= k_{41}k_{43}k_{45}k_{46}k_{50}(k_{48}+k_{52}) \\ \alpha^{\prime} &= k_{41}k_{43}k_{44}k_{50}k_{52}(k_{47}+k_{48}) \\ \beta^{\prime} &= k_{41}k_{43}k_{45}k_{46}k_{48}k_{49}\\ \gamma &= k_{45}k_{50}k_{51}k_{52}(k_{42}+k_{43})(k_{47}+k_{48}). \end{aligned} $$

The relative concentration of *K*_*OO*_ is thus given by the invariant in Eq. 1.

The tree of loops structure of the Ssk1 network has an important consequence. It implies that the steady-state ratio of active to total Ssk1 is independent of the upstream biochemistry (i.e., the mechanistic details of the various Sln1 and Ypd1 reactions) as long as some process exists to generate positive levels of *Y* and *Y*_*P*_. In that case, the ratio will always be given by Eq. 1, although the numerical value will of course differ depending on steady-state concentrations of *Y* and *Y*_*P*_. The implications of this result, including its suggestion that robustness in the HOG pathway is independent of putative Sln1 bifunctionality, are considered in the conclusion.

### Breakdown of robustness due to depletion of the Ypd1 pool

The invariant derived from our mathematical model (Eq. 1) suggests that Ypd1 levels are critical to robustness. The denominator of the invariant is quadratic in the concentration of free *Y*_*P*_ and linear in the concentration of free *Y*. Provided that the upstream network favors production of *Y*_*P*_ over *Y* and that there is substantially more Ypd1 than Ssk1, the denominator of Eq. 1 will be large, and the relative concentration of *K*_*OO*_ will be maintained at a low level. This situation allows for considerable under- or overexpression of pathway components without spurious activation of the HOG pathway, in agreement with our experimental findings.

The invariant predicts that massive overexpression of Ypd1 should not cause phosphorylation of Hog1. In fact, additional Ypd1 would drive the $\frac {[K_{\textit {OO}}]}{K_{T}}$ ratio even closer to zero, lowering the amount of unphophorylated Ssk1 required for HOG pathway activation. In contrast, Ypd1 underexpression should increase the ratio, potentially compromising fitness due to inappropriate activation of the HOG pathway. Similarly, massive overexpression of Ssk1 should deplete free *Y* and *Y*_*P*_ due to increased levels of the four intermediate complexes (*Y*_*P*_*K*_*OO*_, *Y*_*P*_*K*_*OP*_, *YK*_*OP*_, and *YK*_*PP*_). Under the assumption of tight binding between Ypd1 and Ssk1 in each of these complexes, which is well-supported by existing kinetic data [20,22], very little free Ypd1 will be present at steady state if there is much more Ssk1 than Ypd1. As such, Eq. 1 predicts that the ratio will be higher following massive overexpression of Ssk1 than under wild-type conditions.

We experimentally validated these three predictions. The GEV system can achieve at most a 10-fold increase in protein expression from a single inducible allele [11]. We created haploid GEV yeast strains carrying high-copy 2 *μ* plasmids with a GEV-inducible allele (P _*GAL*1_-GENE) of a gene of interest, which allowed us to test the model prediction that massive overexpression of Ssk1, but not of Ypd1, should lead to inappropriate HOG pathway activation. These high-copy yeast plasmids are estimated to be present at 15-50 copies per cell [25-28].

**Figure 5:**
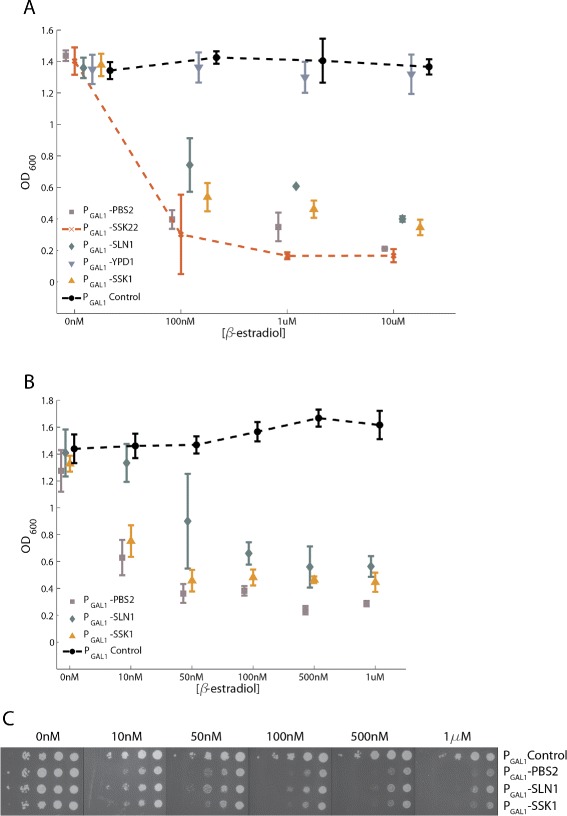
**Massive overexpression of phospho-relay components leads to growth defects.** **A** Haploid GEV strains carrying a high-copy plasmid with an inducible HOG pathway gene were grown in different concentrations of *β*-estradiol. The OD _600_ after 36 hours of growth is plotted as a function of *β*-estradiol concentration. Each point represents the mean and standard deviation of four replicates. Overexpression of Sln1 and Ssk1 (but not Ypd1) caused a growth defect. **B** The same strains were grown over a finer titration of *β*-estradiol concentrations. The OD _600_ after 36 hours is plotted. At this resolution, it is clear that the growth defect from Ssk1 overexpression is more severe than the growth defect from Sln1 overexpression at low *β*-estradiol concentrations. **C** The same strains were frogged onto plates containing different concentrations of *β*-estradiol. Massive overexpression of Sln1 and Ssk1 again caused a growth defect comparable to that from overexpression of Pbs2. In all experiments, the parent strain carrying the empty vector plasmid [2 *μ* *P*_*GAL*1_ scURA3] was used as a negative control.

We measured the growth of these strains in different concentrations of *β*-estradiol to assay for growth defects that might be due to HOG pathway hyperactivation. Massive overexpression of both Sln1 and Ssk1 caused a growth defect over a range of *β*-estradiol concentrations, but the strain with Ypd1 overexpressed grew as well as a wild-type strain carrying only the empty vector (P _*GAL*1_ 2 *μ* scURA3) (Figure 5A). Examination of growth over a finer range of *β*-estradiol concentrations indicated that overexpression of Ssk1 caused a more severe growth defect than overexpression of Sln1 (Figure 5B). These defects were also visible on solid media (Figure 5C). As such, extreme overexpression of Ssk1 compromises fitness.

**Figure 6:**
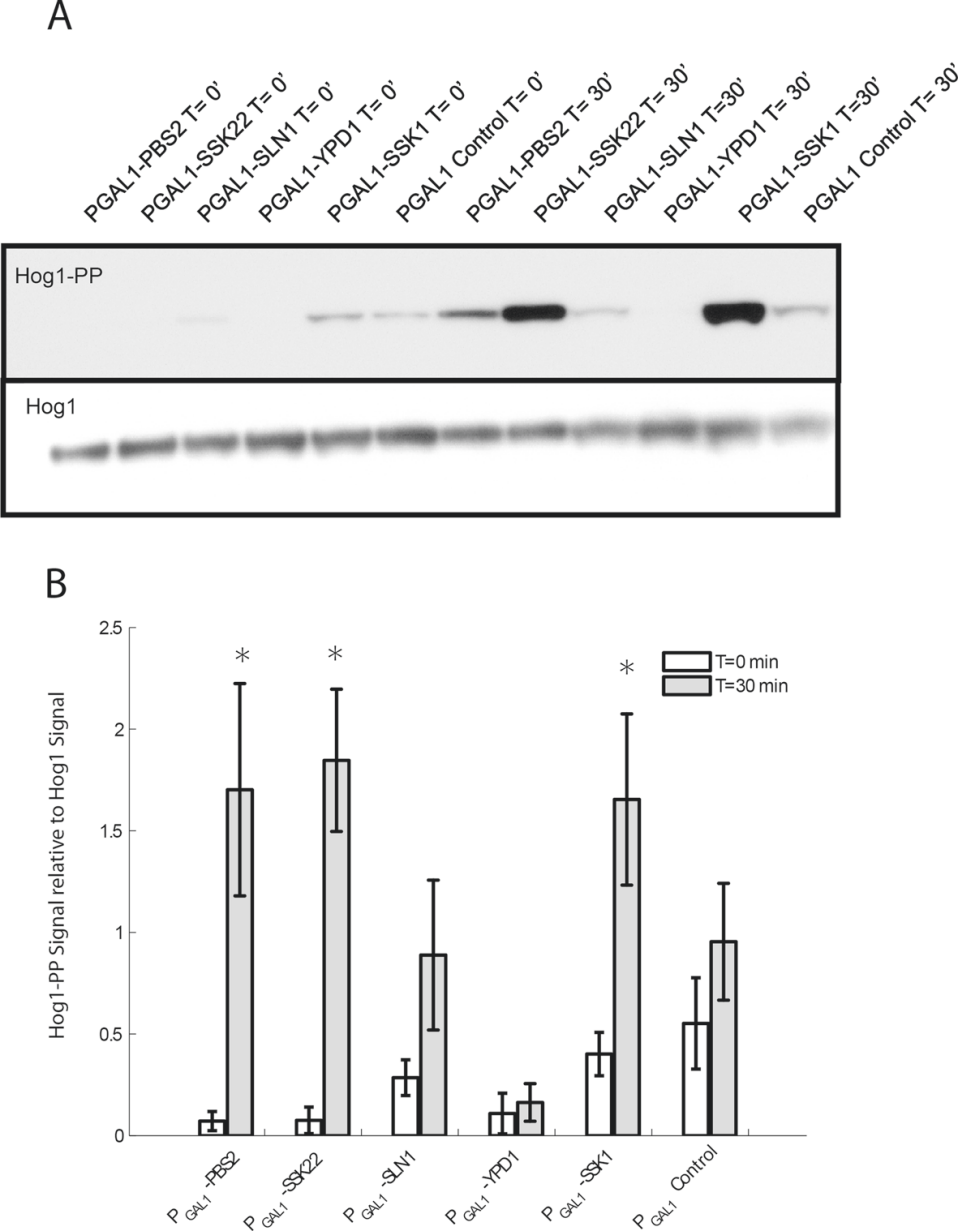
**Growth defects following massive overexpression of phospho-relay components are due to activation of the HOG pathway.** **A** We assayed for Hog1 phosphorylation after overexpression of relay components (Sln1, Ypd1, Ssk1) and positive controls (Pbs2, Ssk22). The parental strain carrying the empty plasmid vector was used as a negative control. **B** We quantified the amount of phosphorylated Hog1 (relative to Hog1) in five biological replicates of this experiment. Error bars represent the standard error. Pbs2, Ssk22, and Ssk1 caused a significant (∗) change in Hog1 phosphorylation levels after overexpression for 30 minutes (*p*=0.0395, 0.0096, and 0.0224, respectively; paired t-test). Hog1 phosphorylation levels were also significantly lower in the Ypd1 overexpression strain (*p*=0.0246, two-way ANOVA).

We again assayed phospho-Hog1 levels to check if overexpression of Sln1 and Ssk1 causes activation of the HOG pathway in normal osmolarity conditions (Figure 6). We measured Hog1 phosphorylation levels after GEV-induction of relay components and of the positive controls Pbs2 and Ssk22 from a multi-copy plasmid. Overexpression of Pbs2, Ssk22, and Ssk1 caused a significant change in Hog1 phosphorylation after 30 minutes (Figure 6B). Although some Hog1 phosphorylation was observed after overexpression of Sln1, the increase was insignificant. Interestingly, overexpression of Ypd1 did not cause an increase in the level of phosphorylated Hog1. In fact, levels of Hog1 phosphorylation were reduced in the Ypd1 overexpression strain (*p*=0.0246, two-way ANOVA).

**Figure 7:**
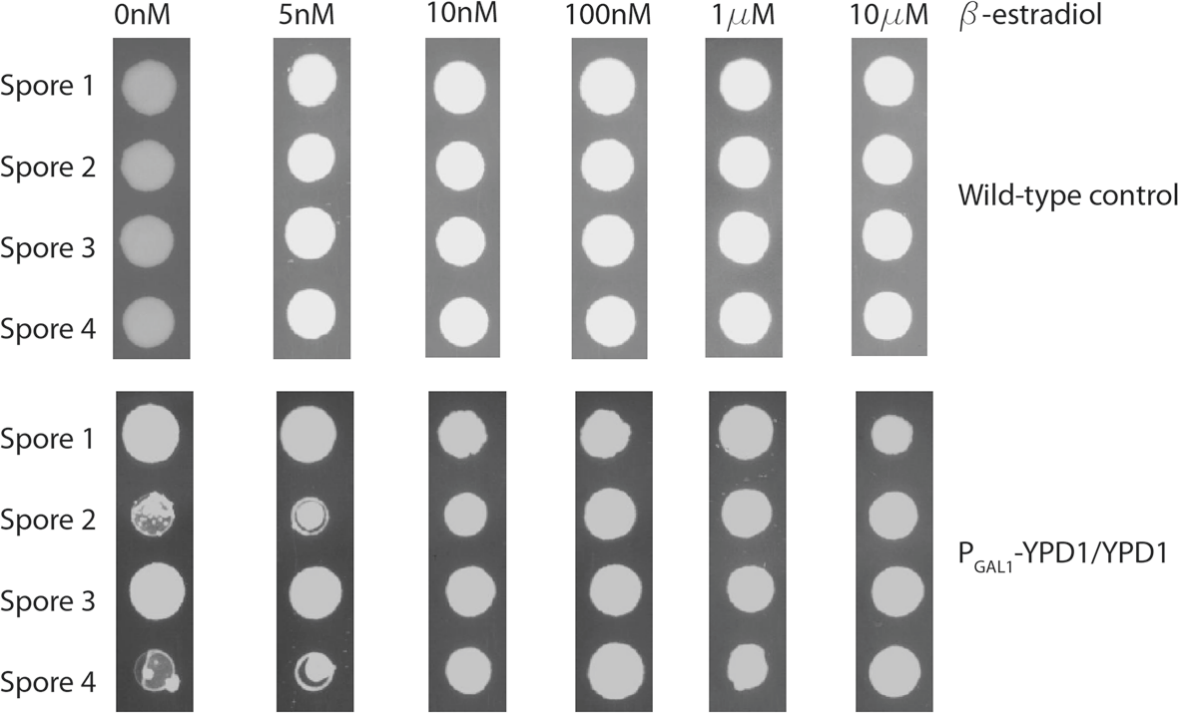
**Underexpression of Ypd1 causes a severe growth defect.** A diploid homozygous GEV strain carrying one inducible allele of Ypd1 (YPD1/P _*GAL*1_-YPD1) was sporulated onto 10 nM *β*-estradiol, and individual spores were frogged onto plates containing different concentrations of *β*-estradiol. At low concentrations of *β*-estradiol, spores carrying the inducible allele (P _*GAL*1_-YPD1, row 2 and row 4) exhibited a growth defect due lower levels of Ypd1 and hyperactivation of the HOG pathway. Spores carrying the wild-type allele (row 1 and row 3) showed no growth defect.

We could not perform underexpression experiments in a ypd1 *Δ* background because deletion of Ypd1 is lethal. To underexpress Ypd1, we instead sporulated the diploid strain (P _*GAL*1_-YPD1/YPD1) containing a wild-type copy of *YPD1* and a single copy under the control of P _*GAL*1_ onto media containing 10 nM *β*-estradiol. We reasoned that 10 nM *β*-estradiol would give sufficient expression of Ypd1 for cell growth, which was confirmed by observation of four viable spores. We then grew these spores on media containing a range of *β*-estradiol concentrations (Figure 7). Ypd1 underexpression caused a clear growth defect compared to the wild-type control on 0 nM and 5 nM *β*-estradiol, indicating that Ypd1 underexpression is toxic. In contrast, underexpression of Sln1 and Ssk1 did not cause a growth defect (Additional file 1: Figure S1).

### The growth defect following Sln1 overexpression is only partially due to HOG pathway activation

As discussed above, we observed a slight but non-significant increase in Hog1 phosphorylation following massive overexpression of Sln1 (Figure 6). We therefore investigated whether the growth defect in response to Sln1 overexpression is only partially due to HOG pathway activation by creating yeast strains null for *SSK1*. Activation of the HOG cascade through the Sln1 branch requires Ssk1, so in ssk1 *Δ* strains it is not possible for Sln1 overexpression to activate the HOG pathway.

**Figure 8:**
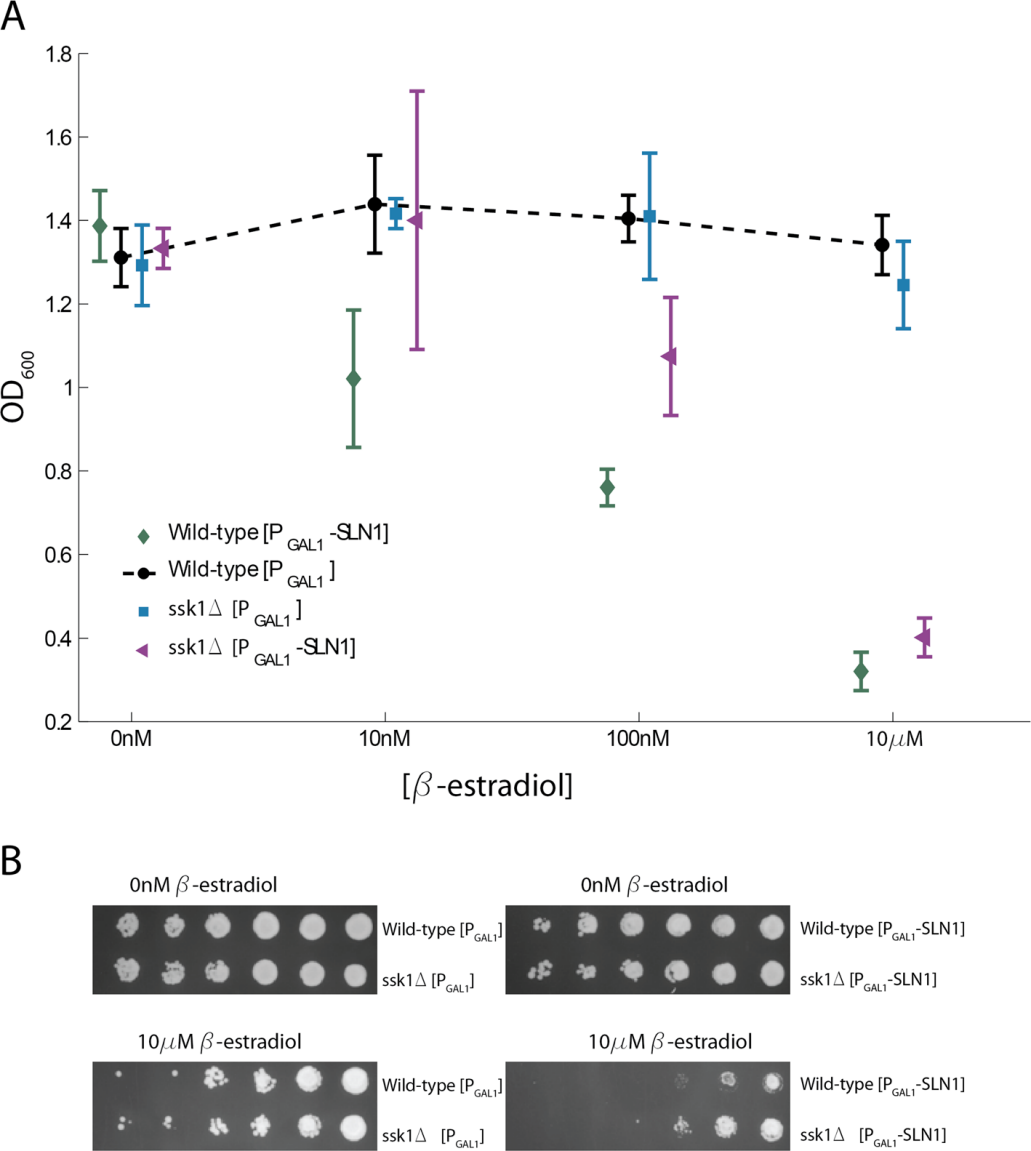
**Growth defects following Sln1 overexpression are not completely due to HOG pathway activation.** **A** We assayed growth in wild-type and ssk1 *Δ* strains overexpressing Sln1 in response to *β*-estradiol or carrying an empty P _*GAL*1_ vector control. Deletion of Ssk1, which prevented HOG pathway activation by Sln1, partially alleviated the growth defect due to Sln1 overexpression. **B** Growth of cells on plates containing 10 *μ*M *β*-estradiol indicated that ssk1 *Δ* reduced the toxicity of Sln1 overexpression.

Overexpression of Sln1 from a 2 *μ* plasmid using GEV was still detrimental to growth in the ssk1 *Δ* strain (Figure 8), indicating that the growth defect due to Sln1 overexpression is only partially due to HOG pathway activation. This result held both for growth in liquid cultures (Figure 8A) and on solid media (Figure 8B). It is consistent with Sln1 overexpression causing a smaller effect on Hog1 phosphorylation levels (Figure 6).

## Conclusions

Robustness of the Sln1-Ypd1-Ssk1 phospho-relay is essential to prevent spurious activation of the HOG pathway, which severely compromises yeast fitness. We established that the phospho-relay is robust to perturbations in the concentrations of the three relay components. A theoretical analysis suggested that a large pool of the intermediate component Ypd1 can buffer fluctuations in other pathway components to maintain robustness. This suggestion was consistent with earlier published measurements indicating that Ypd1 is at least 5 times more abundant than Ssk1 at normal expression levels [7,29]. Although Ypd1 may also bind to the protein Skn7, combined levels of Ssk1 and Skn7 have been measured to be below total Ypd1 levels [29]. Our subsequent experiments confirmed that depletion of this buffering pool of Ypd1 leads to inappropriate activation of the HOG pathway.

The differential expression of Ypd1 and Ssk1 enables phosphorylation of excess Ssk1 and stabilization of the new phospho-Ssk1, buffering HOG pathway activation to fluctuations in Ssk1 levels. This novel mechanism of robustness suggests an advantage of a three-component architecture over a two-component one. In particular, the implementation of an analogous buffering strategy in a two-component system would be difficult because it would require expressing the sensor histidine kinase at very high levels. This situation might lead to imprecise sensing and various other off-target effects. In contrast, the use of an intermediate transfer protein enables robust buffering with both the sensor and response regulator expressed at comparable levels. Our work has thus identified a potential mechanism for circumventing a trade-off between efficient sensing and robust control. There are other possible advantages for a three-component architecture, including combinatorial control of response regulators by sensor proteins through a common phosphotransfer protein or segregation of sensing and activation functions between the nucleus and cytoplasm. Intriguingly, deletion of YPD1 has recently been shown to cause constitutive activation of the HOG pathway in *Candida albicans*, suggesting that its buffering capacity might also be important in this organism [30].

Robustness in real biological systems is necessarily approximate and apt to be compromised at extreme expression levels of cellular components. In many systems, however, it has proven difficult to characterize where robustness breaks down and to reconcile such results with mathematical models, which often predict exact robustness [31]. Our combined theoretical and experimental results specify a single condition (Ypd1 in large excess) for robust regulation of the HOG pathway.

The link between bifunctionality and robustness is well-established [31-35], and it is known that bifunctionality of EnvZ is essential to robustness in *Escherichia coli* osmoregulation [36,37]. As such, it is intriguing that our model suggests that robustness in *S. cerevisiae* osmoregulation is not dependent on bifunctionality of Sln1. It is important to emphasize, however, that bifunctionality would not compromise robustness. Rather, the model indicates that any upstream process that produces non-zero levels of *Y* and *Y*_*P*_ should enable the same fundamental behavior predicted by Eq. 1. The possibility that Sln1 exhibits phosphatase activity warrants further experimental investigation.

## Methods

### Yeast strains and media

All yeast strains used in this study are listed in Additional file 2: Table S1. The homozygous GEV diploid strain, which served as the wild-type background strain for all diploid overexpression experiments, was created by mating haploid GEV strains yMM598 and yMM1101 [11] and picking zygotes to create yMM1104. As described previously [11], diploid yeast strains capable of overexpressing the desired HOG pathway protein from a single locus (yMM1263, yMM1272, yMM1259) were created by transforming [38] the homozygous GEV diploid strain yMM1104 with the KanMX-P _*GAL*1_ cassette amplified from yMM1100 genomic DNA using appropriate oligonucleotide pairs. Transformants were verified by colony PCR and sequencing.

Yeast strains containing 2 *μ* plasmids for massive overexpression of pathway components (yMM1313-yMM1318) were constructed using recombination-mediated plasmid construction [39,40] to generate the overexpression plasmids pMM330-pMM334 *in vivo* (as described below). Positive transformants were selected for and maintained on SC-Ura media [38].

Yeast strains containing the P _*STL*1_-YFP reporter of HOG pathway activity and one estradiol-inducible allele of a HOG pathway gene (yMM1296, yMM1298, yMM1300, yMM1301, yMM1304, yMM1305) were constructed by transforming the heterozygous diploid GEV yeast strains (yMM1104, yMM1272, yMM1264, yMM1259 yMM1286, yMM1287) already containing an inducible allele with the product of PCRing yECitrine-HphMX off plasmid pMM280 using appropriate oligonucleotides. The oligonucleotides contained homology such that the *STL1* ORF was replaced with the yECitrine-HphMX cassette. Transformants were verified by colony PCR, and expression of YFP in 1M sorbitol was assayed.

Yeast strains null for the *SSK1* gene (ssk1 *Δ*) were created by deleting the *SSK1* ORF using appropriate oligonucleotide pairs to amplify KanMX from pMM131 and transforming it into yMM630. Transformants were selected for drug resistance and verified by colony PCR and sequencing.

Standard yeast media was used as noted. Low fluorescence yeast media was prepared as described previously [41].

### Plasmid construction

All plasmids used in this study are listed in Additional file 3: Table S2. Plasmid pMM329 (P _*GAL*1_ scURA3 2 *μ*) was constructed by PCR of the native *GAL1* promoter from genomic DNA prepared from yMM1100 using appropriate primers. This promoter was ligated in pMM12 between the restriction sites KpnI and XhoI (scURA3 2 *μ*) [42]. The resulting plasmid served as a template to create a series of overexpression plasmids with different HOG pathway genes under the control of the *GAL1* promoter using yeast recombination-mediated plasmid construction [39,40]. Appropriate primer pairs were used to amplify Pbs2, Ssk22, Sln1, Ypd1, and Ssk1, respectively, from yMM1100 genomic DNA. These PCR products were then co-transformed with pMM329 linearized with XhoI and SalI. The primer pairs used to amplify the HOG pathway genes contained homology with the pMM329 backbone such that the gene of interest was integrated after P _*GAL*1_ to create a P _*GAL*1_-HOGGENE plasmid (pMM330-pMM334). Positive transformants in which the plasmid had been repaired were selected for on SC-Ura media. Plasmids were purified from these transformants and verified by sequencing.

### Overexpression growth experiments on solid media

Yeast strains were grown overnight to saturation in appropriate media (YPD or SC-Ura media to maintain plasmids). These saturated cultures were serially diluted in 10-fold increments and frogged onto YPD or SC-URA plates containing 0 nM, 10 nM, 100 nM, 1 *μ*M, or 10 *μ*M *β*-estradiol (Tocris Biosciences). These plates were incubated at 30°C for two days before imaging.

### Overexpression growth experiments using plate reader

Yeast strains were grown overnight to saturation in YPD or SC-Ura media. In the morning, each strain was diluted 1:2000 into 200 *μ*L of the same media containing 0 nM, 100 nM, 1 *μ*M, or 10 *μ*M of *β*-estradiol in a well of a 96-well flat-bottom plate (Costar). Each strain/estradiol combination was run in four replicates on the same plate. Growth curves were generated using a Synergy H1 microplate reader (BioTek). Cells were grown at 30°C with continuous, double-orbital (555 cpm) shaking, and OD _600_ was measured every 20 minutes. Growth rates were calculated from the growth curves using spline-fits determined with the R package grofit [43]. OD was plotted at the time points indicated in the figure legends.

### Underexpression experiments by diploid sporulation

Diploid GEV strains yMM1104 (control), yMM1259 (SLN1/KanMX-P _*GAL*1_-SLN1), yMM1263 (SSK1/KanMX-P _*GAL*1_-SSK1), yMM1264 (SSK1/KanMX-P _*GAL*1_-SSK1), and yMM1272 (YPD1/KanMXrev-P _*GAL*1_-YPD1) were sporulated in 1 *%* potassium acetate for 3 days and dissected onto YPD plates containing 10 nM *β*-estradiol. Two spores from each tetrad contained the wild-type HOG pathway gene (Sln1, Ypd1, or Ssk1), while the other contained the same gene under the control of the *GAL1* promoter (KanMX-P _*GAL*1_-SSK1, KanMX-P _*GAL*1_-SLN1, KanMX-P _*GAL*1_-YPD1). After all spores had grown to a sufficient size, they were diluted into YPD and frogged onto YPD plates containing 0 nM, 5 nM, 10 nM, 100 nM, 1 *μ*M, or 10 *μ*M *β*-estradiol. Spores were allowed to grow at 30°C for 2 days prior to imaging.

### *P*_*STL*1_-yEVenus induction and flow cytometry

We created diploid GEV strains that carried both an inducible HOG gene under the control of P _*GAL*1_ promoter and a HOG pathway transcriptional reporter (P _*STL*1_-yEVenus) to assay for downstream transcriptional activation in response to overexpression of various HOG pathway proteins. Strains were grown with agitation in low fluorescence media at 30°C to mid-log (Klett 80), at which point 200 *μ*l of cell culture was sampled for flow cytometry by adding it to 800 *μ*l of cold PBS + 0.1 *%* Tween 20 stored at 4°C. Each culture was induced by adding *β*-estradiol to a final concentration of 10 *μ*M. Cultures were sampled for flow cytometry after induction with *β*-estradiol at *T*=2 hours and *T*=19 hours. Fluorescence was analyzed by flow cytometry on a BD LSRII Multi-Laser Analyzer with HTS (BD Biosciences).

### Preparation of protein extracts

We measured levels of phosphorylated Hog1 following both moderate and massive overexpression of pathway components. For the moderate overexpression experiments, diploid GEV yeast strains yMM1104, yMM1263, yMM1259, yMM1272, and yMM1287, which each contained one estradiol-inducible copy of a HOG pathway gene, were grown to mid-log (Klett 80) in YPD at 30°C with shaking. To assess the effect of massive overexpression of HOG pathway proteins, yeast strains containing the P _*GAL*1_-HOGGENE scURA3 2 *μ* overexpression plasmids (yMM1313-yMM1318) were grown in SC-Ura media to mid-log (Klett 80) at 30°C with shaking. For all strains, at *T*=0 expression of the gene of interest was induced by addition of 10 *μ*M *β*-estradiol (final concentration). At indicated timepoints, 1.5 ml of culture was sampled.

Protein was prepped from samples immediately after each time point. Each sample was centrifuged (1320 RPM) and the supernatant aspirated. The resulting cell pellet was resuspended in 100 *μ*l of 1X sample buffer (Invitrogen) with *β*-mercaptoethanol (final concentration of 10 *%*), protease inhibitor (Roche), and phosphatase inhibitor (Fisher Scientific). Samples were heated at 95°C for 5 minutes, vortexed for 2 minutes, and then rapidly frozen in liquid nitrogen and stored at -20°C.

### Western blotting

Prior to western blotting, samples were thawed and centrifuged at 1320 RPM for 5 minutes. They were run on 4-10 *%* Bis-Tris gels (Invitrogen) and transferred to PVDF membranes (Invitrogen) by electrophoresis at 13 V for 4 hours. Membranes were blocked for 1 hour at room temperature with agitation in 1X TBS, 0.1 *%* Tween-20, and 5 *%* milk. Membranes were then probed with primary antibody overnight at 4°C.

The following antibodies were used to detect phosphorylated Hog1, total Hog1, and actin, respectively: anti-phospho-p38 MAPK rabbit monoclonal antibodies (Cell Signaling Technology *#*9215), anti-c-myc goat polyclonal antibodies (Santa Cruz Biotechnology sc-6815), and anti- *β*-actin antibody (Abcam ab8224). All primary antibodies were diluted 1:1000 in 1X TBS, 0.1 *%* Tween-20, and 5 *%* milk. Following incubation with the primary antibody, membranes were washed (4×5 minutes) with TBST (1X TBS, 1 *%* Tween-20) and then incubated for 1 hour at room temperature with the appropriate secondary antibody conjugated to HRP (anti-rabbit IgG (Cell Signaling *#*7074, 1:5000 dilution), rabbit anti-goat IgG (Santa Cruz sc-2768, 1:5000 dilution), and anti-mouse IgG (Abcam ab97023, 1:20000 dilution), respectively). All secondary antibodies were diluted in 5 *%* milk, 1X TBS, and 1 *%* Tween-20. After incubation with the secondary antibody, membranes were washed 4×5 minutes with 5 *%* milk, 1X TBS, and 1 *%* Tween-20.

Western blots were quantified using chemiluminescence. Membranes were developed using the Pierce Supersignal Femto kit following the manufacturer’s protocol. Chemiluminescence was quantified using HyBlot CL Autoradiography film. Developed film was scanned on a Epson Perfection 4490 Photo Scanner in transmission mode. Protein levels were quantified by densitometry using the Gel Analysis plug-in in ImageJ [44].

Each membrane was probed for phospho-Hog1, beta-actin, and total Hog1 (in that order). After the chemiluminescence assay but before re-blocking, the membrane was stripped by washing in stripping buffer (2×10 minutes), phosphate-buffered saline (2×10 minutes) and 1X TBS + 1 *%* Tween-20 (2×5 minutes). Stripping buffer contained 15 g glycine, 1 g SDS, and 10 mL Tween 20 in 1 L ultrapure water, with the pH adjusted to 2.2 using concentrated HCl.

## Additional files

Additional file 1
**Figure S1.** Sporulations of P _*G**A**L*1_-GENE/GENE diploids to assess phenotype of reduced phospho-relay component concentration.

Additional file 2
**Table S1.** Table of yeast strains used in this study.

Additional file 3
**Table S2.** Table of plasmids used in this study.
