# Supplementary material for: Robust network structure of the Sln1-Ypd1-Ssk1 three-component phospho-relay prevents unintended activation of the HOG MAPK pathway in Saccharomyces cerevisiae
Source: BMC Syst Biol. 2015 Mar 25;9:17. doi: 10.1186/s12918-015-0158-y (PMC4377207; doi:10.1186/s12918-015-0158-y)
Supplement: Additional file 1 — Figure S1. Sporulations of P GAL1-GENE/GENE diploids to assess phenotype of reduced phospho-relay component concentration. [file 12918_2015_158_MOESM1_ESM.pdf]

Supplemental Figure 1

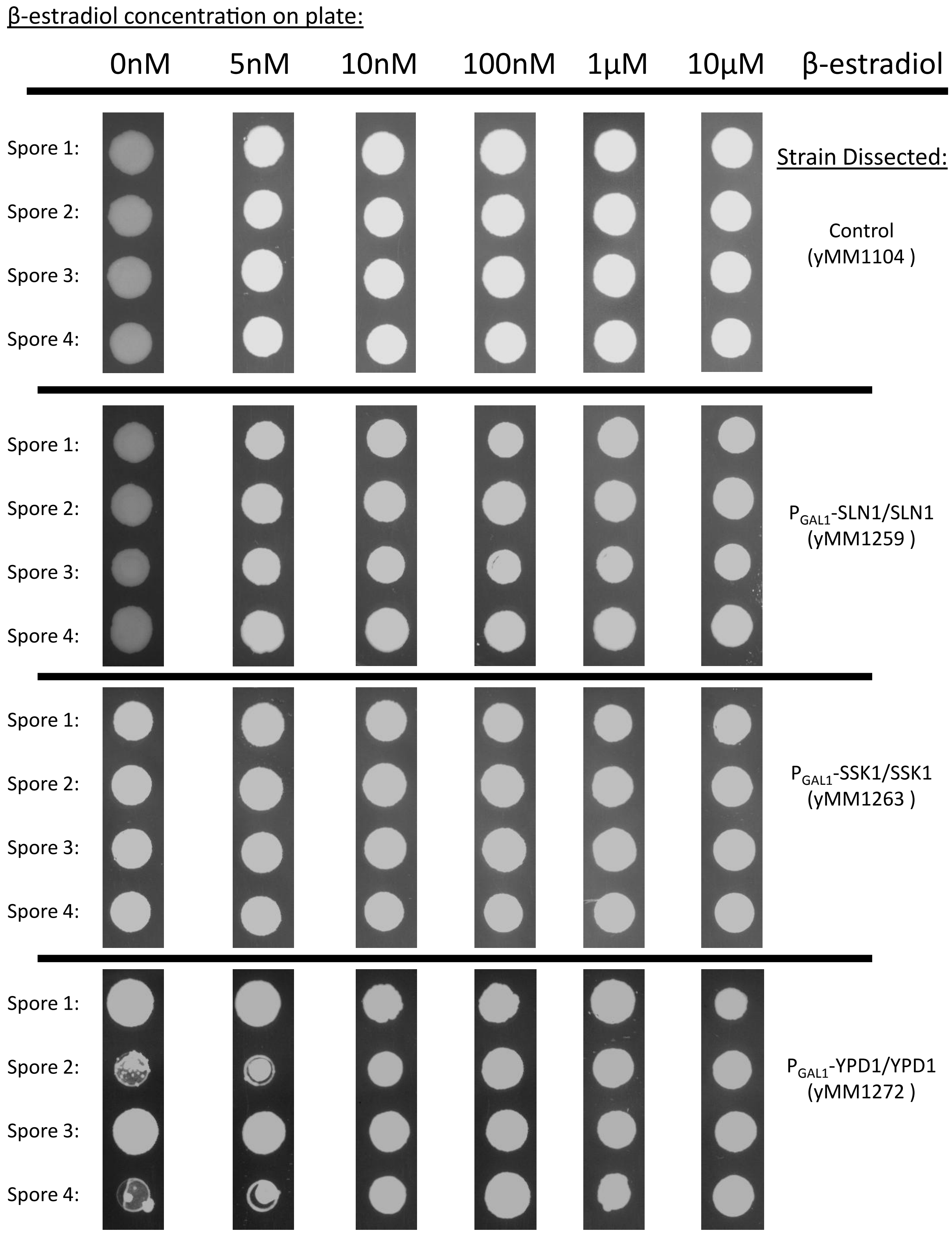

Figure S1: Underexpression of Ypd1 but not other relay components causes a growth defect. Diploid homozygous GEV strains carrying one inducible allele of a relay component (GENE/ PGAL1-GENE) were sporulated onto 10nM β-estradiol and individual spores were frogged onto plates containing different β-estradiol concentrations. Only underexpression of Ypd1 causes a growth defect.
