# Supplementary material for: Robust network structure of the Sln1-Ypd1-Ssk1 three-component phospho-relay prevents unintended activation of the HOG MAPK pathway in Saccharomyces cerevisiae
Source: BMC Syst Biol. 2015 Mar 25;9:17. doi: 10.1186/s12918-015-0158-y (PMC4377207; doi:10.1186/s12918-015-0158-y)
Supplement: Additional file 2 — Table S1. Table of yeast strains used in this study. [file 12918_2015_158_MOESM2_ESM.pdf]

Supplemental Table 1

Strain	Relevant Genotype	Full Genotype	Reference
yMM598	Wild-type GEV Matα	Matα (P_{GAL10}+gal1)Δ::loxP gal4Δ::LEU2 HAP1 leu2Δ0::P_{ACT1}-GEV-NatMX	McIsaac, et al. 2011
yMM630	Wild-type GEV ura3Δ	Matα (P_{GAL10}+gal1)Δ::loxP gal4Δ::LEU2, HAP1 leu2Δ0::P_{ACT1}-GEV-NatMX ura3Δ::HphMX4	McIsaac, et al. 2011
yMM1100	gal10Δ::KanMX	MAT a gal10Δ::KanMX his3Δ1 leu2Δ0 lys2Δ0 ura3Δ0	Invitrogen
yMM1101	Wild-type GEV Mata	Mat a (P_{GAL10}+gal1)Δ::loxP gal4Δ::LEU2 HAP1 leu2Δ0::P_{ACT1}-GEV-NatMX	McIsaac, et al. (DBY12020)
yMM1104	Wild-type GEV/GEV	MATa/α (P_{GAL10}+gal1)Δ::loxP/ (P_{GAL10}+gal1)Δ::loxP leu2Δ0::P_{ACT1}-GEV-NatMX/ leu2Δ0::P_{ACT1}-GEV-NatMX gal4Δ::LEU2/gal4Δ::LEU2	This study
yMM1259	GEV/GEV P_{GAL1}-SLN1/SLN1	MATa/α (P_{GAL10}+gal1)Δ::loxP/ (P_{GAL10}+gal1)Δ::loxP leu2Δ0::P_{ACT1}-GEV-NatMX/ leu2Δ0::P_{ACT1}-GEV-NatMX gal4Δ::LEU2/gal4Δ::LEU2 SLN1/KanMX-P_{GAL1}-SLN1	This study
yMM1263	GEV/GEV P_{GAL1}-SSK1/SSK1	MATa/α (P_{GAL10}+gal1)Δ::loxP/ (P_{GAL10}+gal1)Δ::loxP leu2Δ0::P_{ACT1}-GEV-NatMX/ leu2Δ0::P_{ACT1}-GEV-NatMX gal4Δ::LEU2/gal4Δ::LEU2 SSK1/KanMX-P_{GAL1}-SSK1	This study
yMM1264	GEV/GEV P_{GAL1}-SSK1/SSK1	MATa/α (P_{GAL10}+gal1)Δ::loxP/ (P_{GAL10}+gal1)Δ::loxP leu2Δ0::P_{ACT1}-GEV-NatMX/ leu2Δ0::P_{ACT1}-GEV-NatMX gal4Δ::LEU2/gal4Δ::LEU2 HAP1/HAP1 SSK1/KanMX-P_{GAL1}-SSK1	This study
yMM1272	GEV/GEV P_{GAL1}-YPD1/YPD1	MAT a/α (P_{GAL10}+gal1)Δ::loxP/ (P_{GAL10}+gal1)Δ::loxP leu2Δ0::P_{ACT1}-GEV-NatMX/ leu2Δ0::P_{ACT1}-GEV-NatMX gal4Δ::LEU2/gal4Δ::LEU2 YPD1/KanMX-P_{GAL1}-YPD1	This study
yMM1277	GEV/GEV P_{GAL1}-PBS2/PBS2	MATa/α (P_{GAL10}+gal1)Δ::loxP/ (P_{GAL10}+gal1)Δ::loxP leu2Δ0::P_{ACT1}-GEV-NatMX/ leu2Δ0::P_{ACT1}-GEV-NatMX gal4Δ::LEU2/gal4Δ::LEU2 HAP1/HAP1 PBS2/KanMX-P_{GAL1}-PBS2	This study
yMM1286	GEV/GEV P_{GAL1}-PBS2/PBS2	MAT a/α HAP1+/HAP1+ (P_{GAL10}+gal1)Δ::loxP/ (P_{GAL10}+gal1)Δ::loxP leu2Δ0::P_{ACT1}-GEV-NatMX/ leu2Δ0::P_{ACT1}-GEV-NatMX gal4Δ::LEU2/gal4Δ::LEU2 PBS2/KanMX-P_{GAL1}-PBS2	This study
yMM1287	GEV/GEV P_{GAL1}-SSK22/SSK22	MAT a/α HAP1+/HAP1+ (P_{GAL10}+gal1)Δ::loxP/ (P_{GAL10}+gal1)Δ::loxP leu2Δ0::P_{ACT1}-GEV-NatMX/ leu2Δ0::P_{ACT1}-GEV-NatMX gal4Δ::LEU2/gal4Δ::LEU2 SSK22/KanMX-P_{GAL1}-SSK22	This study
			This study
yMM1296	Wild-type STL1/P_{STL1}-yEVenus	MATa/α (P_{GAL10}+gal1)Δ::loxP/ (P_{GAL10}+gal1)Δ::loxP leu2Δ0::P_{ACT1}-GEV-NatMX/ leu2Δ0::P_{ACT1}-GEV-NatMX gal4Δ::LEU2/gal4Δ::LEU2 HAP1/HAP1 STL1/P_{STL1}-yEVenus-HphMX	This study
yMM1298	STL1/P_{STL1}-yEVenus P_{GAL1}-YPD1/YPD1	MATa/α (P_{GAL10}+gal1)Δ::loxP/ (P_{GAL10}+gal1)Δ::loxP leu2Δ0::P_{ACT1}-GEV-NatMX/ leu2Δ0::P_{ACT1}-GEV-NatMX gal4Δ::LEU2/gal4Δ::LEU2 YPD1/KanMX-P_{GAL1}-YPD1 STL1/P_{STL1}-yEVenus-HphMX	This study
yMM1300	STL1/P_{STL1}-yEVenus P_{GAL1}-SSK1/SSK1	MAT a/α (P_{GAL10}+gal1)Δ::loxP/ (P_{GAL10}+gal1)Δ::loxP leu2Δ0::P_{ACT1}-GEV-NatMX/ leu2Δ0::P_{ACT1}-GEV-NatMX gal4Δ::LEU2/gal4Δ::LEU2 SSK1/KanMX-P_{GAL1}-SSK1 STL1/P_{STL1}-yEVenus-HphMX	This study
yMM1301	STL1/P_{STL1}-yEVenus P_{GAL1}-SLN1/SLN1	MAT a/α (P_{GAL10}+gal1)Δ::loxP/ (P_{GAL10}+gal1)Δ::loxP leu2Δ0::P_{ACT1}-GEV-NatMX/ leu2Δ0::P_{ACT1}-GEV-NatMX gal4Δ::LEU2/gal4Δ::LEU2 SLN1/KanMX-P_{GAL1}-SLN1 STL1/P_{STL1}-yEVenus-HphMX	This study
yMM1304	STL1/P_{STL1}-yEVenus P_{GAL1}-PBS2/PBS2	MAT a/α (P_{GAL10}+gal1)Δ::loxP/ (P_{GAL10}+gal1)Δ::loxP leu2Δ0::P_{ACT1}-GEV-NatMX/ leu2Δ0::P_{ACT1}-GEV-NatMX gal4Δ::LEU2/gal4Δ::LEU2 PBS2/KanMX-P_{GAL1}-PBS2 STL1/P_{STL1}-yEVenus-HphMX	This study
yMM1305	STL1/P_{STL1}-yEVenus P_{GAL1}-SSK22/SSK22	MAT a/α (P_{GAL10}+gal1)Δ::loxP/ (P_{GAL10}+gal1)Δ::loxP leu2Δ0::P_{ACT1}-GEV-NatMX/ leu2Δ0::P_{ACT1}-GEV-NatMX gal4Δ::LEU2/gal4Δ::LEU2 SSK22/KanMX-P_{GAL1}-SSK22 STL1/P_{STL1}-yEVenus-HphMX	This study
yMM1313	[P_{GAL1}-PBS2 scURA3 2μ]	Matα (P_{GAL10}+gal1)Δ::loxP, gal4Δ::LEU2, HAP1 leu2Δ0::P_{ACT1}-GEV-NatMX ura3Δ::HphMX [pMM330 P_{GAL1}-PBS2 scURA3 2μ]	This study
yMM1314	[P_{GAL1}-SSK22 scURA3 2μ]	Matα (P_{GAL10}+gal1)Δ::loxP, gal4Δ::LEU2, HAP1 leu2Δ0::P_{ACT1}-GEV-NatMX ura3Δ::HphMX [pMM331 P_{GAL1}-SSK22 scURA3 2μ]	This study
yMM1315	[P_{GAL1}-SLN1 scURA3 2μ]	Matα (P_{GAL10}+gal1)Δ::loxP, gal4Δ::LEU2, HAP1 leu2Δ0::P_{ACT1}-GEV-NatMX ura3Δ::HphMX [pMM332 P_{GAL1}-SLN1 scURA3 2μ]	This study
yMM1316	[P_{GAL1}-YPD1 scURA3 2μ]	Matα (P_{GAL10}+gal1)Δ::loxP, gal4Δ::LEU2, HAP1 leu2Δ0::P_{ACT1}-GEV-NatMX ura3Δ::HphMX4 [pMM333 P_{GAL1}-YPD1 scURA3 2μ]	This study
yMM1317	[P_{GAL1}-SSK1 scURA3 2μ]	Matα (P_{GAL10}+gal1)Δ::loxP, gal4Δ::LEU2, HAP1 leu2Δ0::P_{ACT1}-GEV-NatMX ura3Δ::HphMX4 [pMM334 P_{GAL1}-SSK1 scURA3 2μ]	This study
yMM1318	[P_{GAL1} scURA3 2μ]	Matα (P_{GAL10}+gal1)Δ::loxP, gal4Δ::LEU2, HAP1 leu2Δ0::P_{ACT1}-GEV-NatMX ura3Δ::HphMX4 [pMM329 P_{GAL1} scURA3 2μ]	This study
yMM1333	ssk1Δ	(P_{GAL10}+gal1)Δ::loxP gal4Δ::LEU2 HAP1 leu2Δ0::P_{ACT1}-GEV-NatMX ura3::HphMX ssk1::KanMX	This study
yMM1335	ssk1Δ [P_{GAL1} scURA3 2μ]	(P_{GAL10}+gal1)Δ::loxP gal4Δ::LEU2 HAP1 leu2Δ0::P_{ACT1}-GEV-NatMX ura3::HphMX ssk1::KanMX [pMM329 P_{GAL1} scURA3 2μ]	This study
yMM1338	ssk1Δ [P_{GAL1}-SLN1 scURA3 2μ]	(P_{GAL10}+gal1)Δ::loxP gal4Δ::LEU2 HAP1 leu2Δ0::P_{ACT1}-GEV-NatMX ura3::HphMX ssk1::KanMX [pMM332 P_{GAL1}-SLN1 scURA3 2μ]	This study

Table S1: Yeast strains used in this study.
