# Supplementary material for: Robust network structure of the Sln1-Ypd1-Ssk1 three-component phospho-relay prevents unintended activation of the HOG MAPK pathway in Saccharomyces cerevisiae
Source: BMC Syst Biol. 2015 Mar 25;9:17. doi: 10.1186/s12918-015-0158-y (PMC4377207; doi:10.1186/s12918-015-0158-y)
Supplement: Additional file 3 — Table S2. Table of plasmids used in this study. [file 12918_2015_158_MOESM3_ESM.pdf]

Supplemental Table 2

Plasmid ID	Alias	Description	Reference
pMM012	pRS426	scURA3 2µ	Sikorski and Heiter, 1989
pMM131	pFA6-KanMX	KanMX	Goldstein and McCusker, 1999
pMM280	yVenus tag	yEVenus HphMX	This study
pMM329	P_{GAL1} Empty	P_{GAL1} scURA3 2µ	This study
pMM330	P_{GAL1}-PBS2	P_{GAL1}-PBS2 scURA3 2µ	This study
pMM331	P_{GAL1}-SSK22	P_{GAL1}-SSK22 scURA3 2µ	This study
pMM332	P_{GAL1}-SLN1	P_{GAL1}-SLN1 scURA3 2µ	This study
pMM333	P_{GAL1}-YPD1	P_{GAL1}-YPD1 scURA3 2µ	This study
pMM334	P_{GAL1}-SSK1	P_{GAL1}-SSK1 scURA3 2µ	This study

Table S2: Plasmids used in this study.
